# Simulating Potential Associated Socio-Economic Determinants With Sustainable Food Security (A Macro-Micro Spatial Quantitative Model)

**DOI:** 10.3389/fpubh.2022.923705

**Published:** 2022-07-14

**Authors:** Mohammad Reza Pakravan-Charvadeh, Cornelia Flora, Haider A. Khan

**Affiliations:** ^1^Department of Agricultural Economics and Rural Development, Lorestan University, Khorramabad, Iran; ^2^Department of Sociology, Iowa State University, Ames, IA, United States; ^3^Department of Economics, Josef Korbel School of International Studies, University of Denver, Denver, CO, United States

**Keywords:** food security, sustainability, socio-economic factors, logistic regression, Iran

## Abstract

Improving sustainable food security status, nowadays, is an important challenge globally, especially in developing countries. The policy goal should be equity—everyone has the same opportunity to be food secure—rather than equality—everyone gets the same subsidy. Since the culture and socioeconomic status within a country vary from region to region, collapsing all areas into a unique region may introduce errors and inaccurate results, as most studies carried out. This study assesses the geographical pattern of association between food security and socioeconomic factors in urban areas in Iran using a nationally and regionally representative household consumption-expenditure survey from 2010 to 2018. The logistic regression model and big data are used to achieve this goal. The results show that a substantial number of households face food insecurity in urban areas in Iran. Also, different geographic regions have various salient factors that affect food insecurity. Aggregation tests confirmed that researchers should estimate separate models for different provinces, states, and districts to assess and monitor the food security status of a country instead of estimating a unique model for the whole of the country. Geographical disparities should be considered as an important issue before suggesting any catch-all policies for a country. The geo-locational factor of households is a key determinant of the association between socioeconomic factors and food security in urban areas in Iran. In sum, the practical suggestions for improving Iranian households' food security in urban areas are as follows: (1) Developing job opportunities for the head of household. (2) Enhancing the potential for self-employment. (3) Facilitating the study of children within households including providing inexpensive uniforms, books, and materials, especially for poor households. (4) Supporting young couples in terms of accessing to financial resources and providing inexpensive essential equipment of home for them; and (5) Introduction of the importance of dietary diversity and different foods which can be cooked by using these food ingredients within a household. Comparative case studies using similar methodologies can test if our results are generalizable.

## Introduction

Food security, which is the access to sufficient food in the present and future time, according to the rapid growth of the population, faces formidable challenges worldwide. International reports show that, nowadays, about one billion people face food insecurity and malnutrition in the world (1, 2). Based on FAO's (3) report, the world population will reach eight billion by 2025, while food production in countries which face a growing population will not increase by the same proportion (1, 4). Therefore, meeting the demands of the growing population is one of the currently most critical issues for the world community (1, 4). Historically, the first concern of food security and food shortage for the growing population is relevant to the period of Thomas Robert Malthus who published a book entitled “An Essay on the Principle of Population” (5). He wrote, “that the increase of population is necessarily limited using subsistence, that population does invariably increase when the means of subsistence increase, and, that the superior power of population is repressed by moral restraint, vice and misery.” In fact, the power of producing subsistence for man is significantly lower than the power of the growing population. After Malthus, about 200 definitions have been proposed for food security in the world (2, 6). Eventually, all of them were approved by the World Food Conference, and the most accepted definition was proposed in November 1996. In the World Food Conference in 1996 and 2009, food security was defined as the physical, social, and economic access of all people to adequate, safe, and nutritious food that provides their dietary needs and food preferences for an active and healthy life (2, 7). The declaration of the conference confirmed the strong incentive of all nations for improving food security and struggling with poverty and hunger (8, 9). Food insecurity status of a country has two primary causes: (1) At the macro-level, directly related to weaknesses of governments and non-support of national and international NGOs and institutions; (2) At the micro-level, the low level of the socioeconomic and cultural status of individuals or households in accessing sustainable livelihoods and proper nutrition (10). Therefore, emphasis on demographic factors such as socioeconomic, cultural, and political aspects of the human lifestyle is crucial for improving livelihood levels and food security status of households and individuals. The importance of attention to such factors for improving the level of food security has been confirmed by several studies. Most of these studies used a logistic regression model as a standard tool to determine the association between socioeconomic factors and food security (Table 1). The logistic regression model is a statistical technique that applies a logistic function to estimate a binary dependent factor (food secure/insecure groups), although many more complex extensions exist (11, 12). Table 1 shows that most of these studies have been done by using cross-sectional data and quantitative model in a specific location. According to geographical disparities of all provinces in a country in terms of dietary habit, consumption and production pattern, transportation, cultural difference, and family structure, assessing and determining factors associated with food security will not be precise without analyzing the possibility of aggregating provincial data into national data (13). In such a situation, prescribing a particular health and nutrient policy for improving food and nutrition security will be possible responding to geographical differences.

**Table 1:** Literature review in the field of determining factors associated with food security.

**References**	**Study location**	**Food security indicator**	**Statistical model**	**Associated factors**
Masuku et al. (10)	District Thungulu	Calorie	Correlation	Age/Marital status/ Education
Mannaf and Uddin (14)	District Bogra	Calorie	Logistic regression	Age/Family size/ Agricultural income
Wu et al. (15)	Taiwan	UHFSSM	Multinomial logit	Age/Income/Family size
Rossi et al. (16)	City Montevideo	ELCSA	Probit model	Education years/Income
Tabrizi et al. (17)	Northwest of Iran	HFSS	Logistic regression	Age/Gender/ Education/Family size/Region/Occupation
Baumhofer et al. (18)	California	Calorie	Simple regression	Marital status/ Ethnicity/Gender
Laraia et al. (19)	North Carolina	HFSSM	Logistic regression	Age/Ethnicity/ Education/Marital status
Harris-Fry et al. (20)	Bangladesh	Dietary Diversity	Logistic regression	Religion/ Land ownership/Spouse education/Home facilities
Tarasuk et al. (21)	Canada	UHFSSM	Multinomial Logit	Income/Head education and age/Marital status/Ethnicity
Omidvar et al. (22)	Tehran	HFIAS	Multinomial logit	Head education/Religion/ Occupation/Gender
Bulawayo et al. (23)	Zambia	Daily meal frequency	Logistic regression	Family size/ Head education and age/Occupation
Rezazadeh et al. (24)	Urmia/Iran	HFIAS	Logistic regression	Head gender and education/Occupation status
Abdollahi et al. (25)	Pakdasht/Iran	UHFSSM	Logistic regression	Head occupation
Álvares and Amaral (26)	Portugal	UHFSSM	Logistic regression	Head gender, age and education
Carter et al. (27)	New Zealand	NZiDep	Logistic regression	Income/Head age, gender and education, marital status
Magaña-Lemus et al. (28)	Mexico	ELCSA	Logistic regression	Head gender, education and income/Language/Agricultural income
Hosseini et al. (2)	Iran	Calorie	Simple regression	Subsidy reform policy/food prices
Abdullah et al. (29)	Pakistan	Calorie	Logistic regression	Head gender, education and occupation/Family asset
Zakari et al. (30)	Southern Niger	HHFS	Logistic regression	Head gender and occupation/Distance from market
Adeniyi and Dinbabo (31)	North West Nigeria	HDDS-FCS	Correlation	Agricultural experience/Land size/Income

*Source: literature review*.

As our knowledge shows, this paper is the first attempt to assess the geographical differences of factors associated with food security by bringing together these different kinds of studies from a variety of distinct disciplines. To achieve the goal, the geographical association between socioeconomics determinants and food security is assessed in all provinces in Iran. The results of the present study contribute to literature from two different aspects. First, evidence of the relative merits of using provincial data is provided to assess food security in the whole of Iran. Second, a small but growing literature is contributed to the association of socioeconomic factors and food security considering geographical disparities to suggest more specific policies for each place to improve food security. The objectives of the study are as follows:

- Assessing the level of food security of Iranian households in urban areas
- Investigating the possibility of aggregating provincial data for estimation of one model for the country instead of several separated models
- Assessing the association between the geographical pattern of socioeconomic factors with Iranian households' food security in urban areas

## Materials and Methods

### Data Resource

The data and information were drawn from the nationally and regionally representative Household Consumption-Expenditure Survey (HCES) conducted by the Statistical Center of Iran (SCI) (13). This survey has been carried out, for the first time, in 1935 by the Iranian National Bank (INB) to calculate the coefficient of food consumption and life cost indicator. After a long stop, the Economic Bureau of the Iranian National Bank has reiterated its viewpoints on the importance of living costs in 23 cities across the country in 1959. Since 1965, the Iranian Central Bank (ICB) is doing this investigation annually in urban areas. Gathering data and information via the questionnaire was initiated by the ICB on a larger scale. The HCES includes the price, expenditure, and consumption of 267 food ingredients by Iranian households. The similar and homogenous ingredients (such as different types of rice, wheat, etc.) were considered as one ingredient (13). Therefore, about 165 food ingredients in several groups were used to calculate calorie intake consisting of cereals (23 ingredients), vegetables (19 ingredients), fruits (21 ingredients), dried fruits (17 ingredients), dairy products and eggs (12 ingredients), legumes (9 ingredients), fish (3 ingredients), poultry (7 ingredients), red meats (9 ingredients), vegetable oils (3 ingredients), animal fats (3 ingredients), sweets (16 ingredients), spices (18 ingredients), and beverage (5 ingredients) within a household (32).

### Study Location

This study is carried out among urban households in all provinces in Iran. Geographically, Iran is located in West Asia, as the second-largest country, and is divided into 31 provinces and a governor administers each province. The largest provinces are Tehran (8.69 million) and Razavi-Khorasan (6.43 million), and the smallest provinces are Ilam (580 thousand) and Semnan (702 thousand). In the present study, HCES of 1,10,500 Iranian households were used in urban areas from 2010 to 2018.

### Food Security Indicator

Many approaches have been designed to assess and monitor food security status (33). They suggested that selecting an appropriate technique for assessing food security status, globally or internally or locally, is directly related to five criteria: 1- **Specific**: they should be precisely and clearly defined and calculate only the phenomenon of interest. 2- **Measurable**: they should be available and reliable. 3- **Achievable/attainable**: the data and information should be collected feasibly in target population: 4- **Relevant**: they should be beneficial for decision-makers to implement the policy and program. 5- **Time-bound**: they should be collected within an appropriate time (33). In the present study, calorie intake was used as an indicator for food security (2, 33–38) based on **SMART** characteristic, although there is increasing realization that this indicator does not necessarily mean proper nutrition (33, 35). Some studies indicated that when researchers have limited data and information to measure food security, using energy requirements is the best option (2, 33, 39). This indicator is also the best choice to assess and monitor food security status at the national or international level (33, 40, 41). A five-step process was used to calculate a food security indicator as follows:

1. Local measurement units were converted into a standard unit of measurement for each consumed food ingredient (42).
2. Food waste (an inedible portion of each food item) was eliminated through a local food waste table [collected by the National Nutrient and Food Technology Research Institute of Iran (NNFTRI)].
3. The calorie content of all food ingredients was calculated via the national food composition table, which was accumulated by the NNFTRI (2000) (42–44).
4. The calorie intake of each household was computed by summing all calorie contents and then divided by 365 for calculating the daily calories consumed by each household (43, 45).
5. To extract the calorie intake of each individual, the adjusted age of members of each household was calculated by an adult equivalent unit per household in Table 2 (42, 46). Computing the adjusted age of members based on their sex and age will lead to the calculation of the real amount of food consumption within a household (45).

**Table 2:** The adjusted age of the members of the household^a^.

**Age groups**	**Male**	**Female**
0–1	0.33	0.33
1–2	0.46	0.46
2–3	0.54	0.54
3–5	0.62	0.62
5–7	0.74	0.70
7–10	0.84	0.72
10–12	0.88	0.78
12–14	0.96	0.84
14–16	1.06	0.86
16–18	1.14	0.86
18–30	1.04	0.80
30–60	1	0.82
Above 60	0.84	0.74

*^a^Aggregate food calories were adjusted by an adult equivalent unit per household (42, 46), because consumed foods of any member based on sex and age are different within a household and adjusting the number of members is necessary. We should not consider a child as an adult to calculate the needed calories within a household. Table 1 shows that, for example, a male child between 2-3 years old should be considered as 0.54 of an adult person. Also, a female child between 2-3 years old should be considered during the calculation of calorie intake per adult as 0.54 of an adult. Table 1 shows that we can only consider male member between 30-60 years old as one adult within a household*.

*Source: Dercon and Krishnan (46) and Gezimu Gebre (47)*.

The difference between daily calorie intake and the minimum calorie requirement (MCR) of each individual during a day was compared to identify the food security status as follows:


(1) $$\begin{array}{r} y_{i}^{*}=y_{i}\text{-}\gamma_{\text{i}} \end{array}$$

Households with a daily calorie intake per adult equivalent below the daily MCR ($y_{i}^{*}\leq 0$) were considered to be food insecure (13, 32). The MCR (threshold amount) per adult was estimated at 2,200 kilocalories per day in Iran based on the age-sex composition of the household by the National Nutrient and Food Technology Research Institute of Iran (NNFTRI) (2).

### Socioeconomic Factors

Socioeconomic factors were identified by reviewing several studies in the literature (10, 14–16, 18, 21, 23, 24, 48–51). Food security status as a dependent factor was categorized into two groups: food secure households = 1 and food insecure households = 0. The income of the head of the household was divided into quantiles. Household dietary diversity scores (HDDs), as households' access to a variety of foods as a proxy for nutrient adequacy, is a qualitative measurement of the status of food consumption. HDDs for each household was calculated by using the percentage of consumed food ingredients from a total of 267 food items (52). All socioeconomic factors which were used in the final analysis are introduced in Table 3.

**Table 3:** The definition of all factors in the final logistic regression model.

**Factor**	**Definition**
**Dependent**	
FS	Food security status (food secure household = 1, food insecure household = 0)
**Independent**	
Income group	Income group (IG1: The first quintile = 1, otherwise = 0), (IG2: The second quintile = 1, otherwise = 0), (IG3: The third quintile = 1, otherwise = 0), (IG4: The fourth quintile = 1, otherwise = 0), (IG5: The fifth quintile = 1, otherwise = 0) **(Reference group** **=** **RG1)**
Size of household	The number of members of household (N <3 = 0, *N*≥ 3 = 1])
Number of students	The number of students within a household (Nedu <2 = 0, Nedu ≥ 2 = 1)
Gender of head	The gender of head of household (Male = 1, female = 0)
Age of head	The age of head of household (Age <40 = 0, Age ≥ 40 = 1)
The status of education	The status of under education of head of household 1 = if the head of household is under education; 0 = if the head of household is not under education
The status of occupation	The status of occupation of head of household 1 = if the head of household is employed, 0 = if the head of household is unemployed
Married status	Marital status of head of household (married = 1, unmarried = 0)
Home status	The status of homeownership (personal home = 1, rental home = 0)
Home size	The size of home (Hsize <83 = 0, Hsize ≥ 83 = 1)
Food expenditure share	The share of food expenditure
HDDS	Household Dietary Diversity Score (Percentage of the consumed goods from the total 267 Goods list)
Agriculture income	The share of income extracted from agricultural activities

*Source: study results*.

### Statistical Model

The logistic regression model was used to determine socioeconomic factors associated with food security status in different provinces in urban areas in Iran. A significance level of 0.05 was used for all factors. In the final model, only two conceivable outcomes are available, either food secure (*y* = 1) or food insecure (*y* = 0), as follows (13, 53):


(2) $$\begin{array}{r} Log\left(\frac{P_{i}}{1\text{-}P_{i}}\right)=b_{0}+b_{i}x_{i}+\varepsilon \end{array}$$

where P_i_, as the dependent variable, is the probability of being food secure. This relation is regressed upon an entire set of determinants (x_i_) that are believed to have an association with Iranian households' food security (54). To interpret the coefficient of the associated factor in the logistic regression model, Odds ratio (OR) was used as follows:


(3) $$\begin{array}{r} Odd=\frac{P_{\text{i}}}{1\text{-}P_{i}} \end{array}$$

In which P_i_ and 1-P_i_ were defined as the probability of occurring and not occurring an event, respectively. There are three different conditions for odds ratio, including OR = 1, when the exposure is not associated with the odds of the dependent factor; OR > 1, the exposure affect the odds of the outcome at the higher level; and OR < 1, the exposure affect the odds of the outcome at the lower level. Before the estimation of the model, the Variance Inflation Factor (VIF) was used to test multi-collinearity among independent variables (55). All the VIFs were <5, demonstrating that the estimated model has little multi-collinearity (55).

### Aggregation Test of Provincial Data

To test the possibility of aggregating the provincial data into one model for the country, a likelihood ratio (LR) test was investigated using Equation 4 (13):


(4) $$\begin{array}{l} LR\left(i+1\right)=-2LL\left(Pooled\;Model\right)\\ \;-\left[\begin{array}{c} 2LL(Province\;1-2LL(Province\;2)\\ \ldots -2LL(Province\;30) \end{array}\right] \end{array}$$

To use Equation 4, two models were estimated. First, a logit model was estimated with an aggregation of all observations. Second, 270 logit models were estimated, for each province annually, and the log-likelihood statistics were calculated and compared with χ^2^ distribution critical value to determine goodness-of-fit, that is, whether we should estimate a model for each province or the entire country.

## Results

### Sample Description

**Figure 1:**
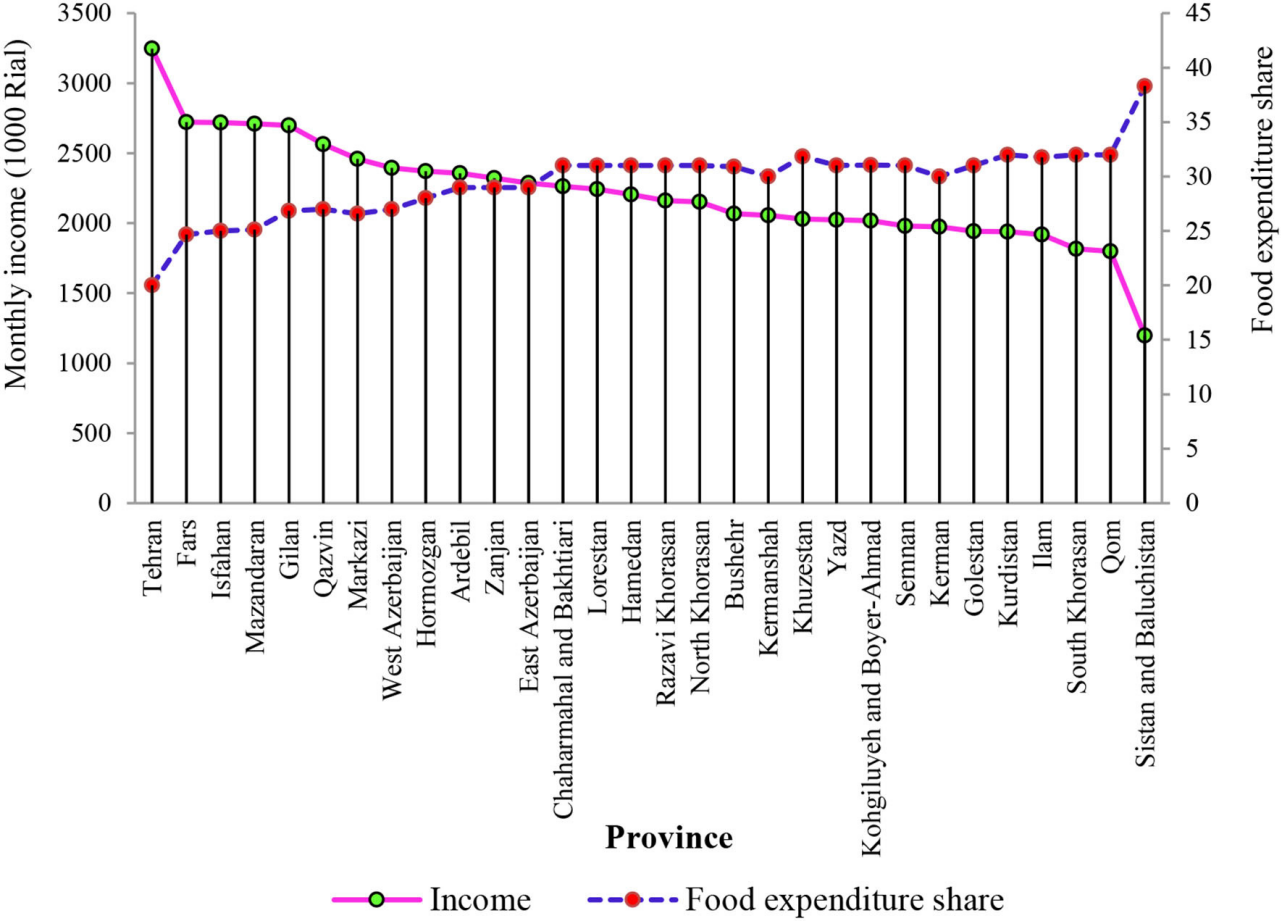
The relationship between monthly income and food expenditure share in all provinces in Iran. Source: study results.

Figure 1 shows the relationship between the household's monthly income and the share of food expenditure. The provinces of Tehran, Fars, and Isfahan had the highest level of monthly income compared to other provinces in urban areas, while the provinces of Sistan and Baluchistan had the least level of income. With a decrease in the level of income from Tehran province to Sistan and Baluchistan, the share of food expenditure increased and therefore, the least and highest level of food expenditure belongs to Sistan and Baluchistan and Tehran provinces, respectively. This result confirms Engel's law in the economic behavior of Iranian households in urban areas. According to this law, which has been proposed by Ernst Engel in 1,857, with the increase in income level of the households, a lower share of the household's total income is allocated to food consumption. This law shows that the income elasticity of food commodities is between 0 and 1 among provinces in Iran.

### Food Security Status

Table 4 shows the status of food insecurity among Iranian households in the urban areas of all provinces. About 41% of Iranian households faced food insecurity in the urban areas. The highest and least percent of prevailing food insecurity were related to Qom (55.9%) and Kermanshah (9.5%) in the urban areas. Over time, the percentage of households who face food insecurity has increased in most of the provinces. As Table 4 shows, the provinces of Yazd and Kuhkiluye and Boyer-Ahmad had a high percentage of food-insecure households on average between 2010 and 2018. About 47% of Iranian households in the urban areas of Tehran province, the most densely populated province in Iran, faced food insecurity and stand on the sixth rank in terms of food security compared to other provinces.

**Table 4:** The percent of food insecure households in urban areas of provinces in Iran.

**Province**	**2010**	**2011**	**2012**	**2013**	**2014**	**2015**	**2016**	**2017**	**2018**	**Average**	**Rank**
East Azerbaijan	16.4	17.4	26.3	20.8	41.5	25.3	27.3	40.5	48.9	29.0	16
West Azerbaijan	7.8	8.8	6.2	6.1	16.6	27.7	35.4	33.3	41.0	20.6	24
Ardebil	9.6	10.6	15.1	10.1	15.7	21.7	15.1	13.9	18.9	15.1	28
Isfahan	28.4	31.6	42.7	38.7	51.6	42.6	46.2	49.2	39.7	42.8	9
Ilam	3.2	4.6	2.3	6.9	7.7	12.8	11.5	6.3	31.7	10.5	29
Bushehr	4.7	5.7	12.8	5.9	47.9	50.2	54.3	70.4	71.7	39.9	10
Tehran	25.1	30.0	43.0	29.5	49.9	54.3	53.9	53.3	66.6	47.6	6
Chaharmahal and Bakhtiari	28.7	37.7	19.9	18.8	53.2	51.5	47.2	35.3	39.2	37.8	12
South Khorasan	4.9	6.4	4.6	2.7	5.7	5.8	30.7	18.7	23.2	9.3	30
Razavi Khorasan	15.1	17.1	12.7	15.8	28.0	31.1	24.9	18.8	17.1	20.7	23
North Khorasan	15.3	17.6	30.3	26.8	32.6	23.0	28.4	30.2	31.4	26.7	17
Khuzestan	12.1	14.1	20.9	21.5	37.1	23.2	17.7	13.2	17.2	19.9	27
Zanjan	7.8	8.9	11.0	20.1	29.2	27.0	29.9	22.7	26.0	21.4	21
Semnan	28.2	32.4	44.1	21.9	57.2	62.0	58.0	55.9	58.0	48.7	4
Sistan and Baluchistan	5.4	7.4	7.2	18.2	26.0	29.8	26.7	28.1	29.1	20.2	25
Fars	4.8	5.9	10.6	16.3	26.1	24.3	32.1	25.1	30.1	21.3	22
Qazvin	16.8	20.4	19.4	20.4	20.2	19.0	26.3	32.5	33.9	24.0	19
Qom	30.5	35.7	38.3	42.8	62.2	55.5	55.0	69.1	70.5	55.9	1
Kurdistan	6.8	7.9	14.4	10.3	26.3	29.7	30.6	25.3	31.3	22.0	20
Kerman	10.3	13.3	9.1	16.0	42.5	37.2	34.2	30.1	28.0	26.3	18
Kermanshah	5.9	6.6	8.2	7.4	7.1	7.7	12.9	13.9	15.0	9.5	31
Kohgiluyeh and Boyer–Ahmad	21.5	23.5	36.9	44.8	57.8	66.2	57.6	55.8	49.6	49.0	3
Golestan	26.0	29.0	26.8	26.4	47.3	53.3	55.5	59.2	55.1	46.1	8
Gilan	24.8	28.8	38.6	35.8	50.3	46.2	57.1	58.0	56.1	46.3	7
Lorestan	9.4	7.2	21.0	10.0	24.9	34.0	53.0	45.7	42.6	29.8	15
Mazandaran	23.9	21.9	37.5	19.5	35.9	41.1	39.8	46.0	59.8	37.7	13
Markazi	10.0	12.0	28.0	22.4	41.3	31.3	44.6	45.2	45.8	32.6	14
Hormozgan	21.4	24.1	31.1	38.4	76.4	65.8	61.6	43.6	44.3	48.2	5
Hamedan	5.9	4.9	16.0	10.4	12.6	21.9	32.9	29.5	32.7	20.1	26
Yazd	42.1	50.0	21.8	35.0	57.7	56.5	59.3	59.2	60.8	54.0	2
**Country**	**35.7**	**35.7**	**35.9**	**42.5**	**43.1**	**40.9**	**41.5**	**43.7**	**45.2**	**41.06**	–

*Source: study results*.

### The Coefficient of Logistic Regression

According to Table 5, because the LR statistics had been greater than the critical value in all provinces, the null hypothesis for the possibility of aggregating provincial data would have been rejected. The logit model should be separately estimated for each of the provinces. In fact, contrary to all studies in the literature which have been carried out in a specific area or state or district, determining factors associated with food security without considering geographical disparities within a country will create deceptive bases for prescribing health and nutrient policies.

**Table 5:** The result of the LR statistic for testing the possibility of aggregating provincial data.

**Province**	**Total**	**2010**	**2011**	**2012**	**2013**	**2014**	**2015**	**2016**	**2017**	**2018**	**LR**
East Azerbaijan	−120	−164	−149	−338	−270	−218	−219	−243	−418	−270	−120
West Azerbaijan	−39	−39	−29	−140	−186	−241	−240	−207	−630	−186	−39
Ardebil	−57	−105	−75	−158	−109	−114	−132	−156	−282	−109	−57
Isfahan	−205	−238	−227	−350	−317	−316	−300	−298	−342	−317	−205
Ilam	−21	−11	−26	−72	−95	−103	−47	−159	−482	−95	−21
Bushehr	−37	−78	−23	−260	−291	−261	−247	−206	−1234	−291	−37
Tehran	−687	−848	−694	−905	−832	−810	−721	−636	−1418	−832	−687
Chaharmahal and Bakhtiari	−110	−99	−100	−232	−243	−205	−175	−214	−528	−243	−110
South Khorasan	−20	−25	−11	−81	−70	−172	−171	−30	−304	−70	−20
Razavi Khorasan	−127	−126	−105	−265	−287	−297	−249	−236	−406	−287	−127
North Khorasan	−74	−126	−152	−383	−336	−324	−330	−256	−478	−336	−74
Khuzestan	−60	−116	−209	−213	−180	−174	−126	−109	−352	−180	−60
Zanjan	−76	−82	−128	−218	−148	−231	−174	−165	−472	−148	−76
Semnan	−121	−324	−114	−195	−193	−175	−186	−173	−532	−193	−121
Sistan and Baluchistan	−72	−59	−190	−252	−187	−224	−268	−186	−698	−187	−72
Fars	−28	−74	−130	−225	−240	−250	−237	−234	−410	−240	−28
Qazvin	−90	−107	−82	−147	−121	−178	−184	−198	−312	−121	−90
Qom	−113	−201	−144	−265	−240	−240	−218	−108	−700	−240	−113
Kurdistan	−63	−96	−64	−173	−187	−170	−165	−187	−620	−187	−63
Kerman	−83	−73	−115	−252	−247	−329	−307	−283	−460	−247	−83
Kermanshah	−59	−72	−65	−128	−95	−138	−134	−113	−238	−95	−59
Kohgiluyeh and Boyer–Ahmad	−86	−103	−125	−225	−209	−286	−230	−216	−866	−209	−86
Golestan	−214	−222	−234	−377	−415	−364	−343	−324	−854	−415	−214
Gilan	−175	−166	−178	−276	−278	−267	−254	−235	−682	−278	−175
Lorestan	−21	−73	−71	−182	−193	−218	−199	−196	−692	−193	−21
Mazandaran	−99	−156	−158	−257	−247	−221	−204	−196	−540	−247	−99
Markazi	−54	−133	−138	−410	−314	−257	−226	−218	−348	−314	−54
Hormozgan	−110	−161	−202	−228	−213	−305	−257	−260	−1176	−213	−110
Hamedan	−32	−92	−70	−186	−271	−306	−286	−275	−134	−271	−32
Yazd	−185	−158	−116	−279	−273	−321	−287	−303	−936	−273	−185

*Source: study results*.

*LR represents Likelihood Ratio*.

Table 6 shows the result of the estimation of the logistic regression models in urban areas among all provinces in Iran. Households who stay in the second to the fifth quintile were more likely to be food secure than those who stay in the first quintile. This association existed among all provinces except Bushehr. Household income was directly associated with food security among urban households in all provinces. Household size was inversely associated with food security in urban areas of 19 provinces in Iran. The probability of prevailing food insecurity will be increased by increasing the number of household members. Households who had at least one child under education as a student at different levels (rudimentary, junior, or senior) were more likely to be food insecure than households without a child under education among 18 provinces. Households whose heads were male were more likely to be food secure than households whose heads were female, including Ardebil, Bushehr, Khuzestan, Zanjan, Kuhkiloye and Boyer-Ahmad, Gilan, and Hamedan, while the status in other provinces was utterly different.

**Table 6:** The results of logistic regression model in urban area of all provinces in Iran.

**Factors**	**Sistan and Baluchistan**	**Semnan**	**Zanjan**	**Khuzestan**	**North Khorasan**	**Razavi Khorasan**	**South Khorasan**	**Chaharmaha and Bakhtiat**	**Tehran**	**Bushehr**	**Ilam**	**Isfahan**	**Ardebil**	**West Azerbaijan**	**East Azerbaijan**
**Income distribution factor**															
Income group 2 = 1, otherwise = 0	1.25*	1.36**	1.30***	2.50***	1.32***	1.63**	1.82***	1.21*	1.94***	0.56	1.09**	0.98	0.95	0.81	1.70***
Income group 3 = 1, otherwise = 0	1.49**	1.66***	1.53***	2.87**	1.88***	1.67***	1.65**	1.36***	1.87***	0.71	0.70	1.52***	1.02**	1.08***	2.28***
Income group 4 = 1, otherwise = 0	2.28***	1.98***	1.96***	2.81**	2.35**	2.19***	2.03***	1.21***	2.04***	0.78	0.76	1.60**	1.33**	1.12***	3.08***
Income group 5 = 1, otherwise = 0	2.01**	2.65***	2.15***	3.63**	3.20**	2.82***	2.31***	1.70***	2.34**	0.74	1.10***	1.60**	1.66**	1.02***	3.26***
**Household's characteristics**															
Size of household	0.90***	0.63*	0.74***	0.68**	0.63***	0.63**	0.49**	0.71*	0.59***	0.66*	0.74	0.80***	0.89***	0.87***	0.88***
Number of students	0.84	0.87**	0.93***	0.95*	0.98***	1.02	1.21	0.84**	0.89**	0.93	0.92*	0.91***	0.87**	0.91***	0.88**
Gender of head	0.67	0.67	1.19**	1.21***	0.86*	0.90**	0.69***	0.83	0.86	1.10***	0.74**	0.97	1.04***	1.33	0.79
Age of head	1.00	1.01	1.01***	1.01	1.00	1.01***	1.01	1	1.01	1.00	1.00	1.01**	1.01***	1.01***	1.01**
The status of education	0.50	0.65	0.76**	0.54	0.60	0.87	0.62***	0.91	0.91***	1.02	0.57	0.84	0.92***	0.68	0.58***
The status of occupation	1.92***	0.51	0.92	1.05**	1.46*	0.61	0.78	0.57	1.16***	0.70	0.55	0.89	1.21**	1.10***	1.08***
Married status	0.92***	0.97	0.74***	1.05	0.73	0.89*	1.55	0.92	0.65***	0.69	1.04	0.73***	0.78	0.66**	0.95***
**Household's assets**															
Home status	0.63	1.27***	1.18***	1.01	1.25*	0.99	0.8	1.17***	1.13**	0.95	1.08***	0.88	1.39**	1.13***	1.09
Home size	1.00	1.00**	1	1.00	1.00	1.00*	1.01	1	1.00***	1.00	1.00**	1.00	1.00	1.00**	1.00
**Household's livelihood factors**															
Food expenditure share	1.02**	1.07**	1.04***	1.06***	1.05**	1.04**	1.03***	1.06**	1.07***	1.05***	1.01*	1.04**	1.03**	1.03**	1.04***
HDDS	1.09***	1.10*	1.08***	1.04***	1.05**	1.10***	1.12***	1.08**	1.08**	1.12***	1.07***	1.07**	1.10**	1.10***	1.07***
Agriculture income	1.01	1.01	1.00***	1.00***	1.01	1.00**	0.99	1.01	1.00	1.01***	0.99	1.01***	1.00***	1.00	1.00***
**Constant**	0.19	0.12	0.1	0.11	0.22	0.22	0.32	0.07	0.08	0.12	7.56	0.09	0.15	0.17	0.13
**Pseudo–R** ^**2**^	0.19	0.2	0.17	0.22	0.20	0.19	0.17	0.21	0.18	0.19	0.20	0.22	0.17	0.22	0.23
**Factors**	**Yazd**	**Hamedan**	**Hormozgan**	**Markazi**	**Mazandaran**	**Lorestan**	**Gilan**	**Golestan**	**Kuhkiloye and Boyer–Ahmad**	**Kermanshah**	**Kerman**	**Kurdistan**	**Gom**	**Qazvin**	**Fars**
**Income distribution factor**															
Income group 2 = 1, otherwise = 0	1.15**	1.08***	1.55***	1.80**	1.30***	1.59**	1.15*	1.21***	0.81	1.19**	0.98	1.22***	1.67*	1.89**	1.31***
Income group 3 = 1, otherwise = 0	1.31***	1.39***	1.44***	2.10**	1.57***	1.84***	1.46**	1.25***	0.89	1.33***	1.27**	1.48***	2.35***	1.74**	1.32***
Income group 4 = 1, otherwise = 0	1.60***	1.66***	1.79***	2.68**	2.15***	1.62***	2.16***	1.43**	1.64***	1.99***	1.43**	1.52**	2.62**	2.03***	1.60***
Income group 5 = 1, otherwise = 0	2.54***	1.83*	2.48***	3.78***	3.24**	1.68*	2.92***	1.38**	3.07*	2.40**	1.69***	2.02***	2.98***	2.29***	1.66**
**Household's characteristics**															
Size of household	0.81	0.72***	0.74	0.78	0.43	0.82	0.59	0.72	0.79	0.83**	0.75	0.72***	0.67	0.88***	0.73***
Number of students	0.81**	1.00	0.86	0.91	0.98**	0.91***	0.90	0.91	0.87	0.92	0.95**	0.98**	0.86	0.87*	0.91***
Gender of head	0.66	1.02***	0.83	0.90***	0.73	0.89***	1.04***	0.66	1.16***	0.83**	0.86	0.87**	0.96***	0.73	0.77
Age of head	1.01	1.01*	1.00	1.01	1.02**	1.01***	1.01***	1.01	1.00	1.01***	1.01***	1.01	1.01	1.01	1.01
The status of education	0.71	0.87***	0.89**	1.04	1.04	0.79***	0.88	0.96***	0.91***	0.82	0.78***	0.64	0.78	0.64	0.77***
The status of occupation	1.33	0.70	0.84	0.93	1.29**	0.80	0.96	1.38***	0.43	0.73	1.08**	1.00	0.94	1.05***	0.73
Married status	1.17	0.68	0.79***	0.77	0.85**	0.71	0.74***	1.05	0.73	1.07	0.85**	0.74	0.53***	0.78**	1.22
**Household's assets**															
Home status	0.82	1.05	1.03	1.13**	1.19**	1.07	1.30***	1.22***	0.73	0.89	0.70	1.10	0.93	1.15**	1.14***
Home size	1.00	1.00***	1.00***	1.00	1.00	1.00	1.00***	1.00	1.00	1.00***	1.00	1.00	1.00	1.00***	1.00
**Household's livelihood factors**															
Food expenditure share	1.03**	1.05**	1.03***	1.04**	1.07*	1.03**	1.06*	1.04***	1.02**	1.03*	1.02***	1.03***	1.06**	1.05**	1.04**
HDDS	1.05***	1.07**	1.07***	1.07***	1.09**	1.10***	1.07***	1.06***	1.07***	1.08**	1.06***	1.08***	1.09**	1.08**	1.09***
Agriculture income	1.01	1.00***	1.01	1.00**	1.01***	0.99	1.01	1.01	1.00***	1.00	1.00	1.00**	1.01	1.01***	1.00***
**Constant**	0.21	0.43	0.09	0.08	0.01	0.08	0.06	0.09	0.23	1.16	0.58	0.22	0.07	0.12	0.15
**Pseudo–R** ^**2**^	0.18	0.19	0.20	0.22	0.18	0.19	0.20	0.17	0.22	0.18	0.20	0.19	0.17	0.21	0.18

*Source: study results*.

*Asterisks three, two, and one are for 1%, 5%, and 10% level of significance*.

On the other hand, households with female heads were more likely to be food secure than those whose heads were male in nine provinces, including Ilam, South Khorasan, Razavi Khorasan, North Khorasan, Qom, Kurdistan, Kermanshah, Lorestan, and Markazi provinces, but not in the others. The age of the head of the households was directly associated with food security in 11 provinces, including East Azerbaijan, West Azerbaijan, Ardebil, Isfahan, Razavi Khorasan, Zanjan, Kerman, Kermanshah, Gilan, Lorestan, Mazandaran, and Hamedan. Household whose head was under training was more likely to be food insecure than household whose head was not under training in 12 provinces, including East Azerbaijan, Ardebil, Tehran, South Khorasan, Zanjan, Fars, Kerman, Kohkiluye and Boyer-Ahmad, Golestan, Lorestan, Hormozgan, and Hamedan, but not in the other 18 provinces. Households whose heads were employed were more likely to be food secure than households whose heads did not have any occupation, including East and West Azerbaijan, Ardebil, Tehran, North Khorasan, Khuzestan, Sistan and Baluchistan, Qazvin, Kerman, Golestan, and Mazandaran, but not in all provinces. Households whose heads were married were more likely to be food secure in 12 provinces consisting of East and West Azerbaijan, Isfahan, Tehran, Razavi Khorasan, Zanjan, Sistan and Baluchistan, Qazvin, Qom, Gilan, Mazandaran, and Hormozgan.

As Table 5 shows, households who own their home were more likely to be food secure than households with a rental home in 14 provinces. The share of food expenditure and dietary diversity had a direct association with food security in the urban areas of all provinces in Iran. Although the share of income extracted from agricultural activities was significantly associated with Iranian households' food security, due to OR = 1 among all provinces, this factor is not considered an important determinant.

## Discussion

The assessment of food security among all provinces in Iran and the associations between socioeconomic factors and food security revealed the importance of geographical diversity for prescribing a specific policy within a country. A three-step process was used. In the first step, the status of food security of Iranian households was assessed in urban areas of all provinces in Iran. As results showed, about 41% of Iranian households faced food insecurity which is not far from the results of other studies (56), by using a systematic review, argued that about 49% of Iranian households faced food insecurity, while 67% of children and 61% of mothers are food insecure (2, 56) showed that almost 32% of Iranian households faced food insecurity from 2007 to 2014 (2). The economic downturn due to the international sanctions has hit the vulnerable poor the hardest (57). Food prices have increased due to implementing subsidy reform policy (2), while the compensatory measures like direct payment to all people equally have not sufficiently covered the necessities of at least half of the Iranian population. Iran is a relatively equal society as measured by the Gini coefficient (57), with a small proportion of the “poor” population. However, a large number of people live just above the poverty line, and they are highly vulnerable to food insecurity shocks (57). It is worth noting that there is a positive outlook on the country's economy if the international sanctions would be lifted. In the wake of the sanctions, most countries, especially adjacent countries, cannot export their foods and agricultural products to Iran, and this restriction creates serious problems for the Iranian people, as shown by the large percentage of food-insecure households.

In the next step, the possibility of aggregating provincial data and information into a unique model for the whole of the country was tested. According to the results, determining socioeconomic factors associated with food in/security without considering spatial and geographical analysis will not yield policies that work across the country. Many studies calculated these factors on a country-side basis, but the results of the present study show the impracticality of prescribing the same health policies in all provinces or states or regions to reduce food insecurity without considering geographical disparities. Before investing in socioeconomic factors to improve food security, as our results confirm, policymakers should determine in which province this factor is significantly associated with food security. Current policies for ameliorating food security are mostly unable to address the issue because in most of the developing countries, especially poor countries, they are suggested for the whole of a country equally, while geographical pattern and the strictness of each region are utterly substantial to introduce different health policies.

In the final step, factors associated with food security were determined in urban areas of all provinces in Iran. Household income was significantly and directly associated with food security as expected. Households whose income was in high quintiles were more likely to be food secure than those in the first quintile. This result is consistent with the results of other studies in the literature (2, 31, 58). Households' income as an access dimension of food security plays a key role in purchasing food ingredients and preparing the needed dietary. Although many developing countries face arduous economic and financial issues, the results confirm that income deciles should be considered as the main factor before implementing a policy to improve Iranian household food security.

The size of households was inversely associated with food security in urban areas of 19 provinces in Iran which is consistent with previous findings (17, 59–62). Increasing the number of household members will increase the fixed and variable expenditure of the family, and therefore, the chance of members accessing adequate food will be decreased. Most family members are consumers in urban areas in Iran. Due to the very high costs of living in urban areas, the chance of larger households is high to be poor (63) and therefore, add more pressure on the size of the households required to feed (64). As results shows, there was no an association between household size and food security in some province. Therefore, it needs some policies emphasizing on the number of household's members for improving food security, and should not be implemented equally among all provinces (23) confirmed a direct positive association between household size and food security in Zambia. They contended that this result might be elucidated by the higher social and productive capital of a larger family (23).

The number of students enrolled in an elementary or secondary school within a household was inversely associated with food security in some provinces. As Iranian households have to pay for the education of their children (including providing uniforms, books, and materials, although schools themselves are free), each additional student within a household increases household food insecurity due to less available money for the food that is consistent with other studies (65, 66). In such a situation, most households relinquish some of their food expenditure to provide their students with what they need to attend school.

The impact of the gender of the head of household on food security varies. Some studies confirmed a positive association between female-headed households and food security (24, 67, 68), while others found that male-headed households were more likely to be food-secure (12, 23, 59). The inverse association between female-headed households and food security may be due to discrimination against women in accessing economic resources (23, 69) and their employment constraints because of household duties. They cannot acquire the needed money for preparing adequate food for the members of the family and hence will be vulnerable to food insecurity. On the other hand, female heads have more knowledge of cooking and preparing high-quality foods as the dimension of the utilization of food security. Therefore, female heads can contribute to their family to improve their food security status.

Households whose heads are students at university levels were more likely to be food insecure in 12 provinces as some studies argued (21), while this association was not significant in others. Although education has vast social benefits, including saving lives through improving households' situations and reducing the risk of conflict (23, 29, 70), when the head of household is enrolled in school, he/she does not have enough time to work full time, reducing household income. Due to the high cost of studying at the superior academic levels in Iran, household heads who are university students have to reduce food expenditure and other expenses to allocate money for studies.

Occupation of the head of the household was directly associated with food security in some provinces, as some studies argued (23, 29), while this factor did not have a significant association in 19 provinces. Occupation of the head of household is a substantial factor in the acquisition of the needed income for feeding all members of a household.

Although home ownership was directly associated with food security in 14 provinces, as some studies explained (71), the size of the home is not associated with food security in all provinces. Households who own their home can save money compared to households who have to pay a lot of their monthly income to rent a home. In such a situation, households have to reduce food consumption to provide housing.

As expected, the share of total household income spent on food was positively associated with food security in urban areas of all provinces in Iran. As food expenditure increases, the probability of acquiring the needed nutrition of family members will be increased and therefore, the probability of being food secure will be enhanced (2) found that a higher share of total expenditures on food leads to higher calorie intake. The same association in all provinces showed that there are no geographical disparities in terms of this factor in Iran (2).

Eventually, the share of consumed food ingredients from all food ingredients was directly associated with food security in urban areas of all provinces in Iran. As the number of consumed food ingredients increases, households have more choices in preparing different foods for their members (2).

## Limitations and Strengths of the Study

The strengths of this study include using big data, population-representative sample, a broad spectrum of socio-economic factors, and examination of geographical disparities of associations between socioeconomic factors and food security. However, the study faced some limitations, including the time-consuming process of calculating the food security indicator and the estimation of a large number of the logistic regression models. Calculation of caloric intake is a limited way to assess food security because of several reasons, including (a) the quality of data of the food supply extracted to assess the indicator can be unreliable, inadequate, and take time to collect (72, 73); (b) according to this index, the average calorie consumption of the target population is tantamount to the average dietary energy supply (33, 74); (c) dietary quality is not considered during the calculation of energy requirement (35); (d) This indicator assumes that calorie intake more than the minimum calorie intake demonstrates the food secure status, but it ignores that some non-communicable disease such as obesity and overweight (caused by inordinate caloric intake) can be a momentous health consequence of excess calorie intake (33, 40). However, due to the national dataset, we did not have any choice in calculating a proxy for food security. Finally, due to the big dataset used in this study, the results may have biases, as other studies have shown (75).

## Policy Implications

Aggregation tests confirm that researchers should estimate separate models for all provinces, states, and districts to assess and monitor food security status in a country, instead of capturing all needed data in the format of a unique model for the whole of the country. With equity of nutritional status as the goal, policies must be designed to fit the situation in diverse areas of food insecurity. Geographical disparities, as results showed, should be considered as an essential issue before suggesting any policy for a country. Geo-location factor of households was found to be a key determinant of an association between socioeconomic factors and food security in urban areas in Iran. Most of the related studies tried to determine factors associated with food security in different districts in the world, but the results of the study confirm that governments can improve the outcomes of implementing different health policies in their countries if they pay attention to distinct dimensions of the effect of geographical factor. Also, the governments, especially in developing countries, should classify all parts of their countries into determined regions based on the common effects of socio-economic factors. Our findings propound the basis for forthcoming studies to classify particular directions for interventions. Our findings reveal that the risk of households' food insecurity also depends on which territory, region, or province they dwell stresses the necessity for more studies to comprehend how practices and policies at this current status of government cope with households' food insecurity prevalence and severity.

## Conclusion

We found that a substantial number of households (about 41%) face food insecurity in urban areas in Iran. Consequently, both government and civil society organizations have important roles to play in addressing this issue. Place-specific policies should be implemented by internal and international institutions and NGOs to reduce prevailing food insecurity. Accessing the full information regarding the food-insecure households along with their dietary habit, characteristics, and geographical disparities can improve the performance of the relevant policy-making institutions, especially the relevant government ministries, to create capabilities to reduce the food insecurity of those households. The government should also consider equitable income distribution as a substantial factor in prescribing any policy. The government should consider the lower income quintile as the target group for the initiation of a policy to ameliorate household food security. A household's income cannot play a key role in improving food security due to the modest association in the provincial models. Therefore, exclusive income enhancement policies cannot be an effective way of improving food security in urban areas of all provinces in Iran. According to the significant association between socioeconomic factors and food security, considering these factors is necessary to prescribe effective policies for improving food security. Different policies will be required in some provinces where these factors are not significantly associated with food security. Therefore, optimal strategies for improving Iranian households' food security in urban areas are as follows:

✓ Developing job opportunities for the head of household;
✓ Enhancing the potential for self-employment;
✓ Facilitating the study of children within households including providing inexpensive uniforms, books, and materials, especially for poor households;
✓ Supporting young couples in terms of accessing financial resources and providing inexpensive essential equipment of home for them;
✓ Introduction of the importance of dietary diversity and different foods that can be cooked by using these food ingredients within a household.

## Data Availability Statement

The raw data supporting the conclusions of this article will be made available by the authors, without undue reservation. The data also is available at https://www.amar.org.ir/.

## Ethics Statement

Ethical review and approval was not required for the study on human participants in accordance with the local legislation and institutional requirements. The patients/participants provided their written informed consent to participate in this study.

## Author Contributions

All authors listed have made a substantial, direct, and intellectual contribution to the work and approved it for publication.

## Conflict of Interest

The authors declare that the research was conducted in the absence of any commercial or financial relationships that could be construed as a potential conflict of interest.

## Publisher's Note

All claims expressed in this article are solely those of the authors and do not necessarily represent those of their affiliated organizations, or those of the publisher, the editors and the reviewers. Any product that may be evaluated in this article, or claim that may be made by its manufacturer, is not guaranteed or endorsed by the publisher.
